# Links among Microbial Communities, Soil Properties and Functions: Are Fungi the Sole Players in Decomposition of Bio-Based and Biodegradable Plastic?

**DOI:** 10.3390/polym14142801

**Published:** 2022-07-09

**Authors:** Vusal Guliyev, Benjawan Tanunchai, Matthias Noll, François Buscot, Witoon Purahong, Evgenia Blagodatskaya

**Affiliations:** 1 Department of Soil Ecology, UFZ-Helmholtz Centre for Environmental Research, 06120 Halle (Saale), Germany; vusal.guliyev@ufz.de (V.G.); tanunchai.benjawan@ufz.de (B.T.); francois.buscot@ufz.de (F.B.); 2 Department of Biology, Leipzig University, 04103 Leipzig, Germany; 3 Institute of Soil Science and Agro Chemistry, Azerbaijan National Academy of Science, Baku 1073, Azerbaijan; 4 Bayreuth Center of Ecology and Environmental Research (BayCEER), University of Bayreuth, 95447 Bayreuth, Germany; matthias.noll@hs-coburg.de; 5 Institute for Bioanalysis, Coburg University of Applied Sciences and Arts, 96450 Coburg, Germany; 6 German Centre for Integrative Biodiversity Research (iDiv), Halle-Jena-Leipzig, 04103 Leipzig, Germany

**Keywords:** PBSA, enzyme activities, bacterial and fungal community composition, bio-based and biodegradable plastic, plastic pollution

## Abstract

The incomplete degradation of bio-based and biodegradable plastics (BBPs) in soils causes multiple threats to soil quality, human health, and food security. Plastic residuals can interact with soil microbial communities. We aimed to link the structure and enzyme-mediated functional traits of a microbial community composition that were present during poly (butylene succinate-co-butylene adipate (PBSA) decomposition in soil with (PSN) and without (PS) the addition of nitrogen fertilizer ((NH_4_)_2_SO_4_). We identified bacterial (*Achromobacter*, *Luteimonas*, *Rhodanobacter*, and *Lysobacter*) and fungal (*Fusarium*, *Chaetomium*, *Clonostachys*, *Fusicolla*, and *Acremonium*) taxa that were linked to the activities of ß-glucosidase, chitinase, phosphatase, and lipase in plastic-amended soils. Fungal biomass increased by 1.7 and 4 times in PS and PSN treatment, respectively, as compared to non-plastic amended soil. PBSA significantly changed the relationships between soil properties (C: N ratio, TN, and pH) and microbial community structure; however, the relationships between fungal biomass and soil enzyme activities remained constant. PBSA significantly altered the relationship between fungal biomass and acid phosphatase. We demonstrated that although the soil functions related to nutrient cycling were not negatively affected in PSN treatment, potential negative effects are reasoned by the enrichment of plant pathogens. We concluded that in comparison to fungi, the bacteria demonstrated a broader functional spectrum in the BBP degradation process.

## 1. Introduction

The use of bio-based plastics is rapidly increasing worldwide because such plastics are increasingly used in the agriculture and food packaging industry [1]. Products of bioplastics are used manifold, for instance, as carrying bags and super-absorbent diapers and for wastewater treatment, various packaging applications, medical and dental implants, catering and hygiene products, and mulching in agriculture. Bio-based plastics are obtained from polymers that are either entirely or partially organic renewable material of biological origin [2]. Unlike traditional plastics, bio-based and biodegradable plastics (BBPs) are metabolized by microorganisms into carbon dioxide (CO_2_) and water under environmental conditions [3]. Therefore, it had been suggested that biodegradable plastics do not impede penetration and the circulation of water and air in soils [4,5]. Although BBPs can degrade in natural environments, especially in soils, their degradation rates vary greatly depending on the type of BBPs, climatic conditions (mainly precipitation and temperature), soil properties, soil types, and organismic diversity as well as activity of soil microorganisms. After one year of exposure in soil, already a 28–33% reduction in bio-based plastic’s poly (butylene succinate-co-butylene adipate) (PBSA) gravimetric and molar mass was found [6]. However, BBPs were not fully mineralized, thus leaving micro- and nanoplastic particles in soils. These scenarios show that BBPs, especially as plastic mulching, can also contribute to the plastic pollution in soil environments. They can also interact with other environmental factors, which may increase risk to plant health. A recent study showed that a high load of microplastics of BBPs interacted with N fertilizer and became a hotspot for an important plant pathogen *Fusarium solani,* which negatively impacted the health of mung bean seedlings [7].

The main advantage of BBPs is the use of renewable resources for their synthesis. The production of many BBPs reduces the carbon (C) footprint compared to petroleum-based plastics [8]. Specifically, BBPs made by plant compounds comprise atmospheric CO_2_ fixed by photosynthesis during cultivation [9]. Many studies have shown that the use of BBPs is more safe to the environment, plants, microorganisms, and humans [5]. Nevertheless, some recent studies have revealed negative effects of BBPs on plant health [10]. The negative effects of BBPs on wheat growth were stronger than those of polyethylene as BBPs inhibited shoot growth and reduced total plant biomass [11]. When soils are contaminated with plant pathogens due to BBPs, the production function is impaired as plants lose their fitness and may not be able to grow and produce biomass.

Although some studies have revealed negative effects of BBPs on crop development and yield prior to harvest, there are still very limited types of BBPs being evaluated in depth. The effect of BBPs on microorganisms is still poorly understood due to applications of low-resolution methods for characterizing microbial communities decomposing plastic and transforming soil organic matter. Healthy soils contain diverse microbial taxa, ensuring ecosystem services such as nutrient cycling and soil fertility [12]. Fungal community members are considered as main decomposers of BBPs especially for PBSA [6,13,14], while less information on the role of bacterial community members in this process is available. Microorganisms present on BBPs added to soils are also frequently associated to atmospheric dinitrogen (N_2_) fixing bacteria (diazotrophs) to receive N supply in N deficient BBP environments [6,15].

Soil microorganisms are pivotal in storing organic C in the soil system [12], regulating the abundance of C in the soil, which in turn affects soil fertility and water storage capacity. The incorporation of C-rich and nutrient-poor substrates such as BBPs into the soil results in a shift of the microbial community structure [16], as BBP-degrading microorganisms, especially fungi, are enriched. Moreover, the addition of BBPs into soil causes increased competitive interactions between plant and soil microorganisms [17]. Many studies have shown no harmful effect of BBP metabolites on soil environments [3,17]. The degradation of BBPs is generally assumed not to negatively affect bacterial biomass and diversity [18], as well as the activity of some soil enzymes [5,17]. However, these findings are based on studies with a limited number of BBP types and soil enzymatic activities. Microbial community structure, richness, and microbial biomass were reported to be good predictors for soil enzyme activities [19]. Some studies have revealed that microbial community structure and enzyme activity in soils are significantly linked with each other, which underlines the microbial structure–function relationships [20,21]. However, some studies also showed that such relationships may not be true in all ecosystems, partly due to the functional redundancy within the microbial communities [22].

Hydrolytic extracellular enzymes such as lipase catalyze the PBSA cleavage into water-soluble compounds [23], which enables the uptake and facilitates soil microbial and plant-microbial interactions [24]. Such hydrolytic enzymes are important for C, N, and P acquisitions and are therefore meaningful indicators for soil functions [25,26]. Lipases are hydrolytic enzymes produced by animals, plants, and microorganisms, which are responsible for the hydrolysis of triacylglycerol into glycerol and free fatty acids [27,28]. Lipases therefore mediate degradation processes of many BBPs [10,29]. Acid phosphatase catalyzes the hydrolysis of organic phosphate compounds to release mineralized P, which increases its bio-availability for uptake by plants and microorganisms [30,31]. N-Acetyl-d-glucosaminidases (NAG) or chitinases are involved in the breakdown of chitin and peptidoglycan, which play an important role in C and N cycling [32]. β-glucosidases catalyze the hydrolysis of cellobiose into glucose [33]. They are often found in soil ecosystems and are considered as a key indicator of soil quality [34].

We aimed to (i) investigate the effects of adding a high load (6% *w/w*) of PBSA to soil with (PSN treatment) and without (PS treatment) the addition of N fertilizer (ammonium sulfate) on important soil parameters (total organic carbon, TOC; total nitrogen, TN; C: N ratio, and pH), fungal biomass, and soil functions (enzyme activities); and (ii) analyze bacterial and fungal communities’ richness, composition, and functional traits, and investigate potential correlations to enzymatic activities (such as lipase, ß-glucosidase, chitinase, and phosphatase). We considered 6% of PBSA as a high plastic load as the maximum plastic contamination in agricultural soil is generally expected to be about 1% [11]. We hypothesized that fungal biomass and soil enzyme activities would increase in both PS and PSN treatments as compared to control soils with and without N addition (control S and control SN, respectively). We expected that a high load of BBPs will change structural and functional relationships between microbial communities, soil properties, and soil enzyme activity in plastic-amended soil as compared to control S and control SN [35,36]. We assumed that fungal community members are not the sole players for the degradation of PBSA, and that bacteria interact with fungi to decompose PBSA.

## 2. Materials and Methods

### 2.1. Experimental Procedure: Soil, PBSA, and Experimental Conditions

In comparison to a published study [10], we further extended the laboratory experiments to link microbial community structure at the family and genus level to enzyme activities, soil pH, ergosterol, and C and N content as affected by the BBP addition. Briefly, we collected soils from a plot under conventional farming and ambient climate treatment at the Global Change Experimental Facility [37], Bad Lauchstadt, central Germany (51°22′60 N, 11°50′60 E, 118 m a.s.l.), which was characterized as a Chernozem with a water holding capacity of 35%, total organic C of 2%, C: N ratio of 10, and pH of 7.5. The conventional farming treatment at GCEF includes a typical regional crop rotation (winter rape, winter wheat, and winter barley) as well as the application of mineral fertilizers and pesticides as described elsewhere [37]. In this study, four soil treatments incubated for 90 d [10] were considered for further analyses: (1) control soil (soil without PBSA) (control S); (2) control soil with (NH_4_)_2_SO_4_ addition (control SN); (3) PBSA–soil with PBSA addition (PS treatment); and (4) PBSA–soil–N soil with PBSA and (NH_4_)_2_SO_4_ addition (PSN treatment). The microbial community composition in the initial soil was also determined in this study along with soil samples from the four treatments and used as a reference. For PBSA–soil treatments (PS and PSN), PBSA films (BioPBS FD92, PTT MCC Biochem Company Limited, Bangkok, Thailand; in the form of double-layer thin film with 50 μm thickness, percent bio-based carbon = 35%) were surface sterilized with 70% ethanol, cut into pieces (2–5 mm × 2–5 mm), 1 g of which was weighed, and buried in a 100 mL sterile glass jar containing 19 g soil (accounting for 15.68 g soil dry weight) from one of the two treatments with five replicates for each treatment and PBSA: dried soil = 6% *w/w*. In control SN and PSN treatments, 1.4 mL of 1.42 M (NH_4_)_2_SO_4_, 0.055 g N, equivalent to 280 kg N per hectare was directly added to the soil to make N available to soil microbes and to mimic fertilization in agricultural systems. For control S and PS treatments, 1.4 mL sterile Milli-Q water was added to achieve a soil water content equivalent to that in PSN treatment (17.5%, accounting for 50% of the water holding capacity), which was considered to be at the field capacity of this soil under actual field conditions. All four soil treatments were incubated at a constant water content of 17.5%, which was determined with a Mettler Toledo HB43-S halogen moisture analyzer (Greifensee, Switzerland) and air temperature at 22 °C for 90 d in the dark, long enough for PBSA to be partially degraded [38]. During the incubation period, the lids of glass jars were manually opened and closed every 14 d under laminar flow to avoid anoxic conditions. After 90 d, PBSA samples were degraded under PS treatment (overall average mass loss = 13%) and highly degraded under PSN treatment (72% in three out of five samples, overall average mass loss = 60%) [39]. A full experimental setup protocol is provided elsewhere [10].

### 2.2. Analyses of Microbial Communities in Soils

Analyses of microbial communities in soils were performed as previously described [10]. Briefly, soil microbiomes were characterized by 16S rRNA gene-based and fungal internal transcribed spacer (ITS)-based amplicon sequencing on the Illumina MiSeq sequencing platform. Soil samples were subjected to DNA extraction using the DNeasy Power-Soil Kit according to the manufacturer’s instructions. After the DNA quantity check, DNA amplification, and visualization by gel electrophoreses, the extracts were subjected to DNA sequencing targeting the 16S rRNA gene V4 region using the universal bacterial/archaeal primer pair 515F (5′-GTGCCAGCMGCCGCGGTAA-3′) and 806R (5′-GGACTACHVGGGTWTCTAAT-3′) [40] and the fungal ITS2 gene using the fungal primer pair fITS7 (5′-GTGARTCATCGAATCTTTG-3′) [41] and ITS4 primer (5′-TCCTCCGCTTATTGATATGC-3′) [42]. Paired-end sequencing (2 × 300 bp) was performed on the pooled PCR products using a MiSeq Reagent kit v3 on an Illumina MiSeq system (Illumina Inc., San Diego, CA, USA) at the Department of Soil Ecology, Helmholtz Centre for Environmental Research, Germany. Additionally, a pooled negative control of all PCR runs was included for sequencing and used as sequencing control. The raw 16S and ITS rRNA gene sequences were deposited in the National Center for Biotechnology Information (NCBI) Sequence Read Archive under the accession number PRJNA702448. After the bioinformatics, we obtained the minimum sequencing depths of 34,856 and 46,400 sequences per sample for the prokaryotic and fungal datasets, respectively. Relative abundance and presence and absence datasets for bacteria and fungi were used in the data analyses. The protocol for the analysis of soil microbiome and all microbial taxonomic and relative abundance information are published elsewhere [10].

### 2.3. Soil Physicochemical Properties and Enzyme Analyses

TOC and TN were analyzed by dry combustion at 1000 °C with an Elementar Vario EL III (Hanau, Germany) elemental analyzer according to DIN/ISO 10,694 (Aug. 1996) [43]. Soil pH was determined using an HI83300 multiparameter-photometer and pH-meter (Hanna instruments, Vöhringen, Germany). The enzyme kinetic parameters (V_max_: maximum rate of enzyme-mediated reactions and K_m_: the concentration of substrate which permits the enzymes to achieve half of V_max_) of β-glucosidase (EC 3.2.1.21), NAG (EC 3.2.1.14), phosphatase (EC 3.1.3.2), and lipase (EC 3.1.1.3) were measured using a fluorometric microplate assay (TECAN Infinite F200Pro, Grödig, Austria) with 4-methylumbelliferone (MUF)-labeled substrates (Sigma-aldrich, Steinheim, Germany). The 4-methylumbelliferyl-beta-D-glucopyranoside (CAS: 18997-57-4), 4-methylumbelliferyl-N-acetyl-beta-D-glucosaminide (CAS: 37067-30-4), 4-methylumbelliferyl-phosphate (CAS: 22919-26-2) and 4-methylumbelliferyl butyrate (CAS: 17695-46-4) were used to detect ß-glucosidase, chitinase, phosphatase and lipase activities, respectively. Soil (0.5 g dry weight equivalent) was sonicated in 50 mL of Milli-Q water for 1 min to make the soil suspension. Additionally, 100 µL of substrate, 50 µL 0.1 M 4-morpholineethane sulphonic acid hemisodium salt [MES (C_6_H_13_NO_4_SNa_0.5_)] (J&K Scientific, Pforzheim, Germany) buffer (pH 6.5) for MUF (C_10_H_8_O_3_) substrate, and 50 µL of soil suspension were added into microplate wells. The time intervals of fluorescence measurements (after 30 min, 90 min, and 150 min) were maintained similarly for all the enzymes and treatments. The enzyme activities were assayed in a range of substrate concentrations (5, 10, 20, 50, 80, 100, 200, and 400 μmol g^−1^ soil). Calibration of fluorometric assay was based on 50 µL of soil suspension (same soil as the soil under study), 50 μL of MES, and in a series of 0–1 mM concentrations of 100 μL of MUF (Sigma-aldrich, Steinheim, Germany, CAS: 90-33-5). Maximal enzyme activities (V_max_) and the concentration of substrate which permits the enzyme to achieve half V_max_ (K_m_) were calculated as released MUF in nmol per g dry soil per h according to the Michaelis–Menten equation [44]. All parameters were modelled with the non-linear regression routine of Origin 2019.

### 2.4. Determination of Ergosterol

To determine ergosterol as an indicator for fungal biomass, 300 mg of fresh soil obtained from 90 d and 1.5 mL of methanol were filled into a 2 mL tube. After 30 s of vortexing with the highest speed (2300 min^−1^), the samples were centrifuged for 5 min at 6217 xg (Centrifuge 5415D, Eppendorf, Hamburg, Germany). The supernatant was passed through a syringe filter (Minisart RC 0.45 µm, Göttingen, Germany) and analyzed in the HPLC Agilent 1100 Series (Agilent, Waldbronn, Germany) using 100% methanol (Merck KGaA, Darmstadt, Germany) mobile solvent equipped with an RP18 150 × 3 mm column according to the manufacturer’s instructions.

### 2.5. Statistical Analysis

As relative abundance data from metabarcoding may contain some biases [45], we analyzed the microbial community composition mainly using presence and absence datasets. The links between microbial community compositions and treatment, soil physicochemical properties, and enzyme activities as well as between microbial communities and enzyme activity patterns were analyzed using the goodness of fit statistic based on presence–absence data and the Jaccard distance measure. The effects of treatments on soil physicochemical properties, microbial traits, and enzyme activities were analyzed using ANOVA and Kruskal–Wallis test for the data with equality and non-equality of variance, respectively. The relationships between bacterial and fungal richness and soil physicochemical properties, microbial traits, and maximum reaction rate of different enzymes (chitinase, lipase, phosphatase, and β–glucosidase) were analyzed using Spearman’s correlation coefficient, incorporating the Jarque–Bera JB test for normality and Levene’s test to assess the equality of group variances. All statistical analyses were performed using PAST version 2.17 [46].

## 3. Results

### 3.1. Bacteria and Fungi in Soils without PBSA and Soils of PBSA–Soil Systems: Who Is Who?

**Figure 1:**
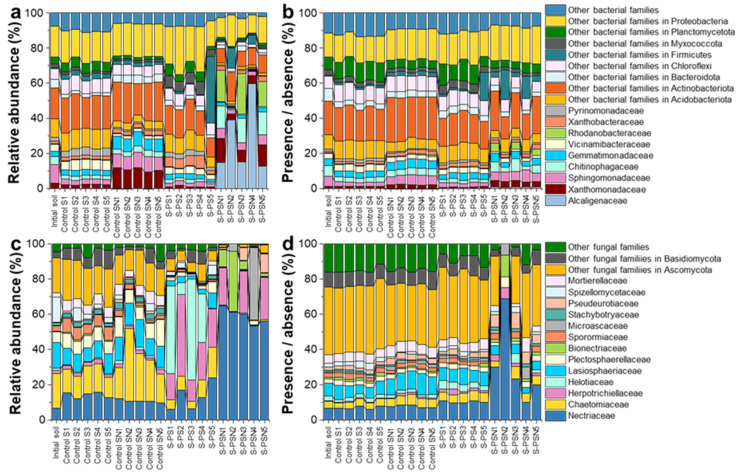
Composition of the bacterial (**a**,**b**) (family level, considering only families with relative abundances ≥ 2%, the rest of the bacterial families were pooled as “others”) and the fungal (**c**,**d**) (family level, considering only families with relative abundances ≥ 1%, the rest of the fungal families were pooled as “others”) communities associated with the degradation of a bio-based and biodegradable poly (butylene succinate-co-adipate) (PBSA) based on relative abundance (left panel, (**a**,**c**)) and presence/absence data (right panel, (**b**,**d**)). Data are presented for initial soil (initial S), control soils (control S), control soils with (NH4)_2_SO_4_ addition (control SN), soils with PBSA addition (S–PS), and soils with PBSA and (NH4)_2_SO_4_ addition (S–PSN).

**Figure 2:**
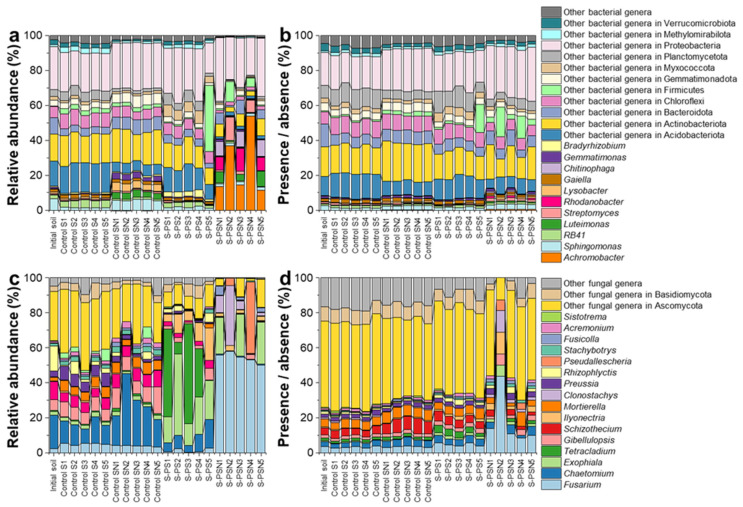
Composition of the bacterial (**a**,**b**) (genus level, considering only families with relative abundances ≥ 1%, the rest of the bacterial genera were pooled as “others”) and the fungal (**c**,**d**) (genus level, considering only families with relative abundances ≥ 1%, the rest of the fungal genera were pooled as “others”) communities associated with the degradation of a bio-based and biodegradable poly (butylene succinate-co-adipate) (PBSA) based on relative abundance (left panel, (**a**,**c**)) and presence/absence data (right panel, (**b**,**d**)). Data are presented for initial soil (initial S), control soils (control S), control soils with (NH4)_2_SO_4_ addition (control SN), soils with PBSA addition (S–PS), and soils with PBSA and (NH4)_2_SO_4_ addition (S–PSN).

The relative abundance data at the phylum and class level of soil microbes were previously published [10]. In this current study, we focused on bacterial and fungal community compositions based on both relative abundance and presence and absence data, which were briefly presented to visualize the most dominant bacterial and fungal families (Figure 1) and genera (Figure 2) for each treatment. We detected 9 and 13 dominant bacterial and fungal families with relative abundances of > 2% and 1%, respectively (Figure 1). The microbial families of the PS and PSN treatments significantly differed from those under the control S and control SN treatment, especially when considering relative abundance. In soils of PSN treatment, *Alcaligenaceae* and *Nectriaceae* were dominant and their relative abundance reached up to 60% and 64%, respectively. In soils of PSN treatment, *Nectriaceae* revealed the highest amplicon sequence variant (ASV) richness compared to other families. The dominant patterns of *Alcaligenaceae* and *Nectriaceae* in soils of PSN treatment correlated to the bacterial genus *Achromobacter* and the fungal genus *Fusarium* (Figure 2).

### 3.2. Effects of PBSA and N Addition on Soil Properties, Fungal Biomass

**Figure 3:**
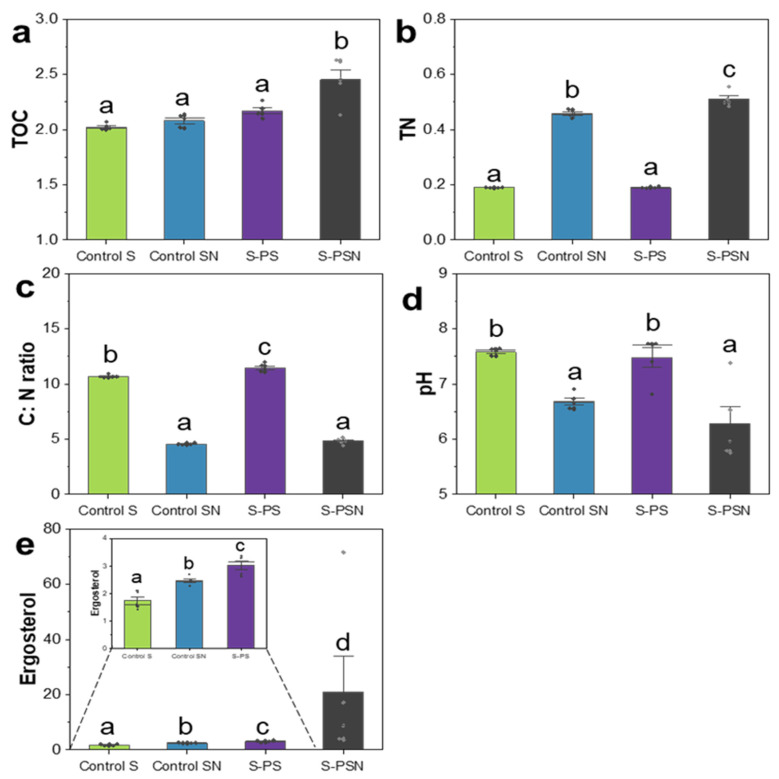
Mean of total organic carbon (TOC, (**a**)), total nitrogen (TN, (**b**)), C: N ratio (**c**), soil pH (**d**), and ergosterol content (**e**) of each treatment. Analysis of variance (ANOVA) or Kruskal–Wallis test was performed for the data with equality and non-equality of variance, respectively. Standard deviation of five replicate measurements are shown. The rhombs on the bars indicate the data points. Different letters indicate significant differences according to ANOVA (*p* < 0.05). Details of the treatment abbreviations can be found in the legend of Figure 1.

The TOC content increased in soils of PSN (2.45%) treatment by 21, 18, and 13%, respectively, as compared with control S (2.02%), control SN (2.08%), and PS (2.17%) treatments (Figure 3). TN was 142% and 169% times higher in soils of control SN (0.46%) and PSN (0.51%) treatments as compared with the control S (0.19%) and PS (0.19%) treatments, respectively (Figure 3). In accordance, the C: N ratio and pH were significantly lower in soils of control SN (4.53 and 6.68) and PSN (4.81 and 6.28) treatments (*p* < 0.05). Fungal biomass based on ergosterol content was the highest in soils of PSN (21.02 mg/kg) treatment (Figure 3), followed by PS (3.04 mg/kg), control SN (2.47 mg/kg), and control S (1.74 mg/kg).

**Figure 4:**
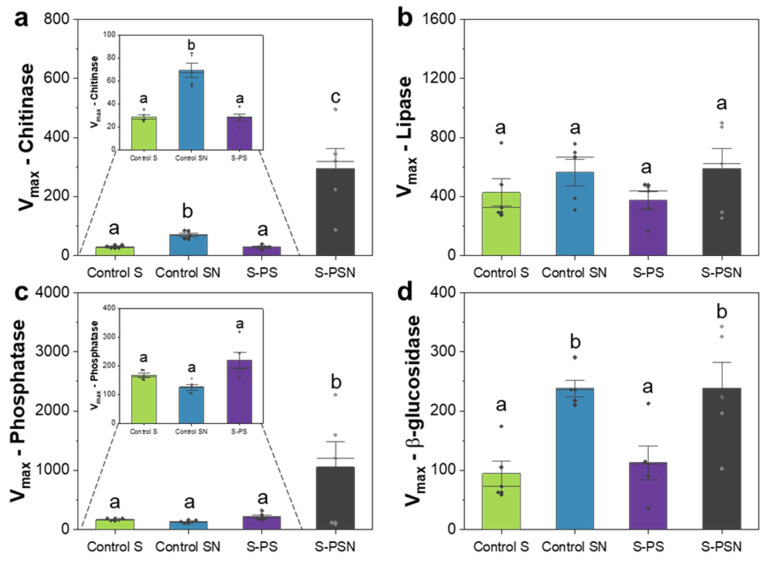
Mean of maximum rate of reaction (V_max_) of chitinase (**a**), lipase (**b**), phosphatase (**c**), and β–glucosidase activity (**d**) of each treatment. Analysis of variance (ANOVA) or Kruskal–Wallis test was performed for the data with equality and non-equality of variance, respectively. Standard deviation of five replicate measurements are shown. The rhombs on the bars indicate the data points. Different letters indicate significant differences according to ANOVA (*p* < 0.05). Details of the treatment abbreviations can be found in the legend of Figure 1.

The enzymatic activities (V_max_) in PS and PSN treatments were enzyme-specific (Figure 4). V_max_ of chitinase and phosphatase reached the highest value in soil under PSN treatment (Figure 4a,c). V_max_ of β–glucosidase was significantly higher in soils with N addition (control SN and PSN treatments) as compared to those without N addition (control S and PS treatments) (Figure 4d).

### 3.3. Microbial Communities Are Shaped by Soil Physicochemical Properties and Linked to Soil Functions

Soil bacterial and fungal community compositions were shaped by the respective treatments (control S and control SN, PS, and PSN) and the soil properties (TOC, TN, C: N ratio, and pH) (Table 1). The main factor that significantly shaped the fungal community composition was TOC (*R*^2^ = 0.88, *p* = 0.001) (Table 1). The enzyme activity was also significantly linked with the microbial community compositions (Table 2). V_max_ of all tested enzymes as well as the K_m_ of phosphatase were significantly correlated to both bacterial and fungal community compositions. Among these enzyme activities, V_max_ of chitinase (*R*^2^ = 0.71, *p* = 0.003) and phosphatase (*R*^2^ = 0.90, *p* = 0.001) were highly correlated with the bacterial community composition, while only V_max_ of chitinase (*R*^2^ = 0.91, *p* = 0.001) correlated with the fungal community composition (Table 2). When comparing control with PBSA-added soils, we found that different bacterial and fungal genera were highly correlated (*ρ* > 0.80, *p* < 0.01) with respective soil functions in control and PBSA-added soils (Table 3). The exception was found for *Achromobacter* and chitinase activity, which were highly correlated both in soils and PBSA–soil systems.

### 3.4. Enzymes Activity Patterns, Soil Properties, and Fungal Biomass: Are There Any Links?

The soil enzyme activity patterns were shaped by treatments, PBSA addition, soil properties (TOC and pH), and fungal biomass (Table S1). The strongest correlation was found between TOC and soil enzyme activity patterns (*R*^2^ = 0.62, *p* = 0.003) (Table S2).

### 3.5. Relationships between Microbial Richness and Soil Properties: Significant Differences between Soils and PBSA–Soil Systems

**Figure 5:**
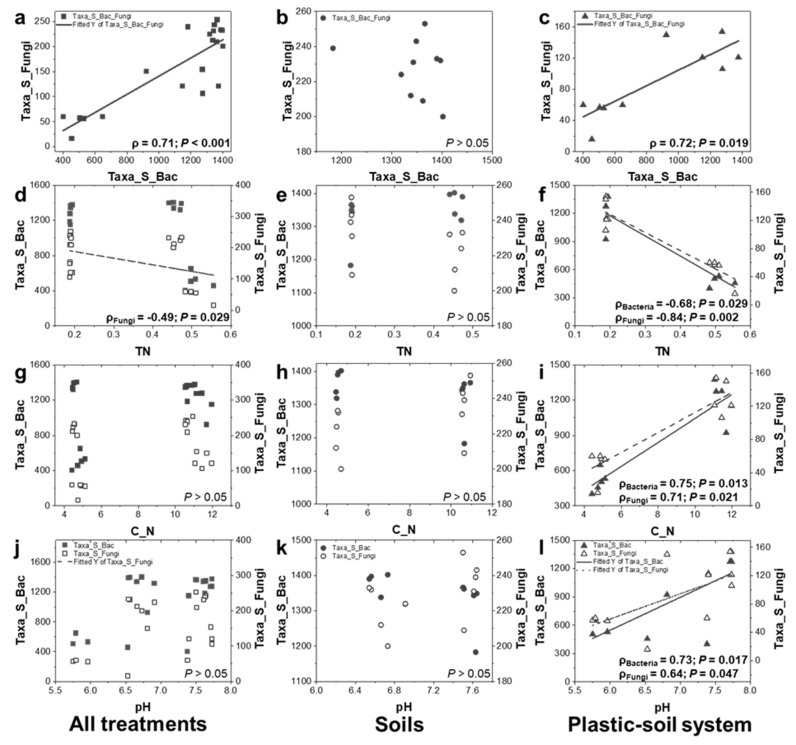
Correlations between bacterial and fungal richness (**a**–**c**), bacterial and fungal richness and total nitrogen (TN) (**d**–**f**), C: N ratio (C_N) (**g**–**i**), and pH (**j**–**l**) in soils of all treatments (left panel, (**a**,**d**,**g**,**j**)), control soils (middle panel, (**b**,**e**,**h**,**k**)), and PBSA-added soils (right panel, (**c**,**f**,**i**,**l**)). Spearman’s rank correlation was performed for aforementioned comparisons. The correlation table for all comparisons is provided in Supplementary Table S1a–c. Statistical significance is given in bold.

Bacterial and fungal richness were significantly positively correlated when considering all treatments (control S, control SN, PS and PSN treatments) together and only in PBSA-added treatments (soils of PS and PSN treatments) (Figure 5a–c). The bacterial and fungal richness corresponded to TN, C: N ratio, and pH differently when considering all treatments, control treatments (control S and control SN), and PBSA-added treatments. When all treatments were considered, no significant correlations were observed between bacterial and fungal richness and TN, C: N ratio, and pH except between fungal richness and TN (Figure 5d). When considering only soils without PBSA addition, no measured soil physicochemical properties (TN, C: N ratio, and pH) were found to shape the bacterial and fungal richness (Figure 5e,h,k). In contrast, when PBSA-added soils were analyzed, both bacterial and fungal richness significantly correlated with TN, C: N ratio, and pH (Figure 5f,i,l).

### 3.6. Relationships between Fungal Biomass and Soil Functions: Consistent for C and N Cycles

**Figure 6:**
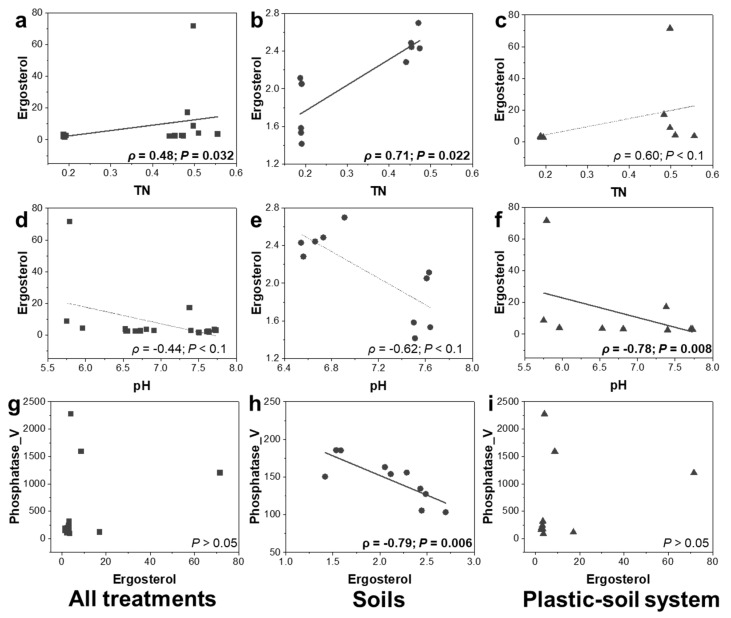
Correlations between total nitrogen (TN) (**a**–**c**), pH (**d**–**f**), maximum rate of reaction (Vmax) of phosphatase (Phosphatase_V) (**g**–**i**), and ergosterol content in soils of all treatments (left panel, (**a**,**d**,**g**)), control S and control SN treatments (middle panel, (**b**,**e**,**h**)), and PS and PSN treatments (right panel, (**c**,**f**,**i**)). Spearman’s rank correlation was performed for aforementioned comparisons. The squares, circles and triangles indicate data points from combination of all treatments, soil without PBSA addition and PBSA added soil respectively. The correlation table for all comparisons is provided in Supplementary Table S1a–c. Statistical significance is given in bold. Thin line indicates marginally significant correlation.

The significant negative correlation between V_max_ of phosphatase and ergosterol content was detected only in soils without PBSA addition, while no correlations were found in all treatments and soils with PBSA addition(Figure 6g–i).

### 3.7. Relationships between Ergosterol Content and Soil Properties

The trend in the correlations between TN, pH, and ergosterol content was consistent across all treatments (Figure 6). We detected positive correlations between TN and ergosterol across all treatments (Figure 6a–c); however, the correlation between ergosterol and TN content was only marginally significant in PBSA-treated soil. Consistent negative correlations were found between ergosterol and pH (Figure 6d–f).

## 4. Discussion

### 4.1. Presence of PBSA Alters Link between Bacterial and Fungal Richness and Its Relationships with Soil Properties

We observed no correlation between bacterial and fungal ASV richness in soil without PBSA addition, whereas in soil with PBSA addition, we detected strong positive correlations (Figure 5). We previously reported such a relationship between bacteria and fungi at the surface of PBSA films in agricultural field soils [6]. In the current study, we demonstrated that this scenario even occurs in the PBSA-added soils. PBSA is degraded as a result of microorganisms’ metabolic activities [47]. Biodegradation of PBSA is considered as an interactive process mediated by different microbial taxa where fungi are characterized as main decomposers, whereas bacteria only contribute as facilitators [6,10,14,48]. Diazotrophs are listed among the most important bacterial facilitators, as PBSA is an extremely N-poor substrate [6]. Nevertheless, some bacteria were also able to directly degrade biodegradable PBSA [49].

The relationships between microbial richness and soil parameters were strongly altered in PBSA-added compared to control soils. In the short term, a high load of N increased the richness of archaeal, bacterial, and fungal taxa in PBSA-added soil [6]. The richness of both bacteria and fungi strongly declined in soil with N and PBSA addition [10]. This was coincident with an increase in soil N content, decrease in C: N ratio, and a reduction in soil pH by approximately ~1–2 units. This may imply that our soil system lost the buffer capacity to maintain microbial diversity against N and pH changes after PBSA addition.

### 4.2. Soil Nutrient Cycling Is Still Functioning despite the High Load of PBSA but How about Soil Health?

In a broad sense, soil functions are the C turnover, plant growth support, water storage, microbial ecosystem functioning, and nutrient cycling [50,51]. Functions of a soil microbial community is the decomposition and transformation of organic matter, which constitutes a transient nutrient sink [52,53]. The widely distributed β-glucosidase enzyme in soil is considered as a key indicator of soil quality and is directly related to the quality and quantity of soil organic matter [34]. Soil functions related to nutrient acquisition through an activity of hydrolytic enzymes were not impaired by the high load of PBSA and were even stimulated in the PSN treatment. The relationships between microbial communities with soil enzyme activities were also highly conserved for the enzymes involved in C and N cycling [54]. However, the soil in PSN treatment was highly governed by broad host range of the plant pathogen *F. solani* [7], which can support the soil nutrient cycling function as it is known as a saprotroph [55]. *F. solani* as well as other *Fusarium* spp. are also well-known to efficiently produce the plastic-degrading enzyme lipase [56], β–glucosidase [57], and chitinase [58], indicating that these species are potent PBSA degraders that are capable to outcompete other PBSA degraders by pathogenic interactions.

### 4.3. Functional Redundancy, Competitions, and Degradation Efficiency

The changes in microbial community composition and reduction in fungal richness in PS and PSN treatments did not affect V_max_ of all measured enzymes (Figure 4). This can be related to functional redundancy of soil microorganisms involved in soil nutrient cycling [59,60,61]. Different microbial communities can process the same soil function as it has been shown in many studies; specifically, different altered community structures and changing domination of key microbial players do not impair main soil functions under environmental conditions [59,60,61]. In our case, we reported before that fungal richness declined in PBSA-amended treatments as compared to control and that the key fungal players were *Tetracladium* spp. and *Exophiala equine* in PS and *F. solani* in PSN treatments [10]. This decline did not negatively impact enzyme-related soil functions (C, N, and P cycling) (Figure 4). Less diverse fungal communities with efficient decomposers were found to have a higher degradation rate of complex substrates due to their reduced investment in fungal–fungal competition [62]. Furthermore, the majority of energy and resources in such a system can be invested in the production of hydrolytic enzymes acquiring nutrients [62,63]. Declined fungal diversity induced by presence of PBSA, however, might reduce the levels of functional redundancy, and as a result, the system may be prone to disturbances [61]. On the other hand, systems containing diverse microbial communities can have a higher possibility to increase levels of functional redundancy [61,64]. There is competition in such systems, which can reduce their efficiency to utilize energy and resources; however, there may also be synergy effects. Importantly, such systems are more resistant or resilient to disturbances [64].

## 5. Conclusions

Our work indicated that the consequences of PBSA application in agricultural soils have to be separately considered for indicators of soil nutrient functions and soil health. Fungal communities significantly corresponded to V_max_ of chitinase and phosphatase, whereas bacterial community composition significantly corresponded to the activity of all measured enzymes. We identified specific bacterial (*Achromobacter*, *Luteimonas*, *Rhodanobacter*, and *Lysobacter*) and fungal (*Fusarium*, *Chaetomium*, *Clonostachys*, *Fusicolla*, and *Acremonium*) genera indicative for PBSA decomposition as their relative abundances were highly correlated with the measured enzyme activities. We also revealed a broader multi-functionality of bacteria versus fungi in degradation of bio-based and biodegradable plastics.

## Figures and Tables

**Table 1:** Goodness of fit statistics (*R*^2^) of treatment and mean of the soil physicochemical parameters fitted to the nonmetric multidimensional scaling (NMDS) ordination of bacterial and fungal community composition based on presence/absence data and Jaccard distance similarity of soils in all treatments. Bold *p* values indicate statistical significances *p* < 0.05.

	Bacteria		Fungi	
	*R* ^2^	*p*	*R* ^2^	*p*
Treatment	0.60	**0.001**	0.54	**0.001**
Total organic carbon (TOC)	0.68	**0.001**	0.88	**0.001**
Total nitrogen (TN)	0.45	**0.001**	0.43	**0.004**
C: N ratio	0.30	**0.034**	0.32	**0.015**
pH	0.58	**0.003**	0.34	**0.016**
PBSA amendment	0.33	**0.033**	0.41	**0.003**
N amendment	0.33	**0.001**	0.31	**0.013**

**Table 2:** Goodness of fit statistics (*R*^2^) of the mean of respective enzyme’s activity fitted to the nonmetric multidimensional scaling (NMDS) ordination of bacterial and fungal community composition based on presence/absence data and Jaccard distance similarity of soils in all treatments. Bold letter indicates statistical significances. The abbreviations are V_max_: maximum rate of reaction of enzymes and K_m_: the concentration of substrate which permits the enzymes to achieve half of V_max_. Bold *p* values indicate statistical significances *p* < 0.05.

	Bacteria		Fungi	
	*R* ^2^	*p*	*R* ^2^	*p*
V_max, Chitinase_	0.71	**0.003**	0.91	**0.001**
V_max, Lipase_	0.42	**0.008**	0.08	0.522
V_max, Phosphatase_	0.90	**0.001**	0.47	**0.020**
V_max, β–Glucosidase_	0.35	**0.024**	0.15	0.249
K_m, Chitinase_	0.04	0.759	0.05	0.640
K_m, Lipase_	0.14	0.215	0.18	0.181
K_m, Phosphatase_	0.61	**0.003**	0.48	**0.008**
K_m, β–Glucosidase_	0.23	0.121	0.12	0.348

**Table 3:** Correlations between relative abundances of bacterial and fungal genera with enzyme activities in control (S) and PBSA-added (*p*) soils. Strong positive correlations above *ρ* > 0.8 are highlighted in green. Bold *p* values indicate statistically significant correlations *p* < 0.05.

Microbial Taxa	β-Glucosidase (*p*)	β-Glucosidase (S)	Chitinase (*p*)	Chitinase (S)	Lipase (*p*)	Lipase (S)	Phosphatase (*p*)	Phosphatase (S)
**Bacteria**								
*Achromobacter*	0.37	**0.87**	**0.81**	**0.89**	0.07	0.20	−0.10	−0.74
*Sphingomonas*	0.02	**0.73**	−0.04	**0.77**	−0.04	0.16	−0.08	−0.43
*RB41*	−0.58	−0.77	−0.68	−0.95	−0.36	−0.33	−0.22	0.59
*Luteimonas*	**0.82**	**0.67**	**0.82**	**0.78**	0.62	0.55	0.44	−0.72
*Streptomyces*	0.18	**0.65**	**0.68**	0.53	−0.12	0.38	−0.27	−0.58
*Rhodanobacter*	**0.80**	0.50	0.59	0.37	**0.81**	0.22	**0.81**	−0.23
*Lysobacter*	−0.39	**0.79**	−0.45	**0.93**	−0.37	0.52	−0.33	−0.68
*Gaiella*	−0.47	0.14	−0.68	0.33	−0.13	−0.26	0.03	−0.21
*Chitinophaga*	**0.75**	0.05	0.54	−0.08	**0.77**	0.07	**0.77**	−0.38
*Gemmatimonas*	0.26	**0.71**	−0.08	**0.75**	0.47	0.48	**0.70**	−0.84
*Bradyrhizobium*	−0.62	0.59	−0.83	0.50	−0.49	−0.03	−0.38	−0.39
**Fungi**								
*Fusarium*	**0.68**	−0.76	**0.92**	−0.64	0.28	−0.25	0.08	0.62
*Chaetomium*	−0.31	**0.79**	−0.76	**0.85**	−0.02	0.38	0.12	−0.56
*Exophiala*	0.42	−0.13	−0.08	0.05	0.61	0.60	0.55	−0.10
*Tetracladium*	−0.58	0.60	−0.84	**0.64**	−0.21	0.35	0.01	−0.53
*Gibellulopsis*	−0.47	0.55	−0.65	**0.78**	−0.24	0.26	−0.26	−0.21
*Schizothecium*	−0.19	0.55	−0.66	0.43	0.10	0.13	0.19	−0.77
*Ilyonectria*	−0.35	−0.85	−0.65	−0.68	−0.25	−0.21	−0.18	**0.68**
*Mortierella*	−0.43	0.19	−0.76	0.03	−0.19	−0.48	−0.18	0.13
*Clonostachys*	**0.82**	−0.68	**0.94**	−0.55	0.44	0.09	0.25	0.36
*Preussia*	−0.34	−0.83	−0.80	−0.68	−0.07	−0.42	0.01	0.50
*Rhizophlyctis*	−0.17	−0.16	−0.64	−0.50	0.12	−0.55	0.18	0.25
*Stachybotrys*	−0.44	0.59	−0.68	**0.65**	−0.20	0.32	−0.24	−0.52
*Fusicolla*	**0.74**	**0.88**	0.20	**0.89**	**0.84**	0.39	**0.85**	−0.58
*Acremonium*	−0.29	**0.81**	−0.71	**0.64**	−0.04	0.19	0.17	−0.50
*Sistotrema*	−0.17	−0.41	−0.17	−0.73	−0.06	−0.79	−0.06	**0.63**

## Data Availability

The raw 16S and ITS rRNA gene sequences were deposited in the National Center for Biotechnology Information (NCBI) Sequence Read Archive under the accession number PRJNA702448.

## Supplementary Materials

The following supporting information can be downloaded at: https://www.mdpi.com/article/10.3390/polym14142801/s1, Table S1: Spearman’s rank correlation between microbial richness, fungal biomass, soil physicochemical properties, and maximum rate of enzyme-mediated reactions (V_max_) of measured enzymes in a) all treatments (control S, control SN, soils of PS and PSN treatments), b) soils without PBSA (control S and control SN), and c) PBSA-added soils (soils of PS and PSN treatments); Table S2: Goodness-of-fit statistics (*R*^2^) of treatment, physicochemical properties, and fungal biomass fitted to the nonmetric multidimensional scaling (NMDS) ordination of enzymes based on Euclidean distance similarity of soils in all treatment. Bold values of *P* and *R*^2^ indicate statistical significances (*p* < 0.05 and *R*^2^ > 0.7).
