# Supplementary material for: Links among Microbial Communities, Soil Properties and Functions: Are Fungi the Sole Players in Decomposition of Bio-Based and Biodegradable Plastic?
Source: Polymers (Basel). 2022 Jul 9;14(14):2801. doi: 10.3390/polym14142801 (PMC9323189; doi:10.3390/polym14142801)
Supplement: Supplementary file 1 [file polymers-14-02801-s001.zip › polymers-1802972-supplementary.pdf]

Supplementary material

Links among Microbial Communities, Soil Properties and Functions: Are Fungi the Sole Players in Decomposition of Bio-Based and Biodegradable Plastic?

Vusal Guliyev [1,2,3,†], Benjawan Tanunchai [1,4,†], Matthias Noll [4,5], François Buscot [1,2,6], Witoon Purahong [1,*] and Evgenia Blagodatskaya [1,*]

[1] Department of Soil Ecology, UFZ-Helmholtz Centre for Environmental Research, Halle (Saale), 06120,Germany
[2] Department of Biology, Leipzig University, Leipzig, 04103, Germany
[3] Institute of Soil Science and Agro Chemistry, Azerbaijan National Academy of Science, Baku, 1073, Azerbaijan
[4] Bayreuth Center of Ecology and Environmental Research (BayCEER), University of Bayreuth, Bayreuth, 95447, Germany
[5] Institute for Bioanalysis, Coburg University of Applied Sciences and Arts, 96450 Coburg, Germany
[6] German Centre for Integrative Biodiversity Research (iDiv), Halle-Jena-Leipzig, Leipzig, 04103, Germany
* Correspondence: witoon.purahong@ufz.de (W.P.); evgenia.blagodatskaya@ufz.de (E.B.)
† These authors contributed equally to this work.

Table S1 Spearman's rank correlation between microbial richness, fungal biomass, soil physicochemical properties, and maximum rate of enzyme-mediated reactions (V_{max}) of measured enzymes in a) all treatments (control S, control SN, soils of PS and PSN treatments), b) soils without PBSA (control S and control SN), and c) PBSA-added soils (soils of PS and PSN treatments).

a) All treatments

	Taxa_S_Bac	Taxa_S_Fungi	TOC	TN	C_N	pH	Ergosterol	Chitinase_V	Lipase_V	Phosphatase_V	ß-glucosidase_V
Taxa_S_Bac		**0.000**	**0.010**	0.240	0.890	0.192	**0.000**	0.128	0.659	0.431	0.955
Taxa_S_Fungi	**0.71**		**0.000**	**0.029**	0.957	0.110	**0.000**	**0.033**	0.925	0.293	0.282
TOC	**-0.56**	**-0.85**		**0.024**	0.715	**0.026**	**0.000**	**0.015**	0.466	0.173	0.105
TN	-0.28	**-0.49**	**0.50**		**0.000**	**0.000**	**0.032**	**0.000**	0.254	0.478	**0.003**
C_N	-0.03	-0.01	0.09	**-0.74**		**0.008**	0.631	**0.002**	0.450	**0.010**	**0.030**
pH	0.30	0.37	**-0.50**	**-0.78**	**0.57**		0.052	**0.000**	0.070	0.927	**0.000**
Ergosterol	**-0.74**	**-0.87**	**0.79**	**0.48**	-0.11	-0.44		**0.003**	0.470	0.235	0.084
Chitinase_V	-0.35	**-0.48**	**0.53**	**0.86**	**-0.66**	**-0.80**	**0.62**		0.079	0.885	**0.000**
Lipase_V	0.11	0.02	0.17	0.27	-0.18	-0.41	0.17	0.40		0.095	**0.003**
Phosphatase_V	-0.19	-0.25	0.32	-0.17	**0.56**	0.02	0.28	-0.03	0.38		0.645
ß-glucosidase_V	0.01	-0.25	0.37	**0.63**	**-0.49**	**-0.78**	0.40	**0.76**	**0.62**	0.11	

b) Control soils

	Taxa_S_Bac	Taxa_S_Fungi	TOC	TN	C_N	pH	Ergosterol	Chitinase_V	Lipase_V	Phosphatase_V	ß-glucosidase_V
Taxa_S_Bac		0.726	0.187	0.511	0.987	0.128	0.987	0.214	0.467	0.701	0.098
Taxa_S_Fungi	-0.13		0.293	0.108	0.162	0.385	0.229	0.467	0.751	**0.022**	0.200
TOC	0.45	-0.37		**0.025**	0.310	0.060	0.174	**0.043**	0.054	0.098	0.082
TN	0.24	-0.54	**0.70**		**0.001**	**0.006**	**0.022**	**0.019**	0.467	**0.009**	**0.025**
C_N	0.01	0.48	-0.36	**-0.89**		**0.029**	**0.009**	**0.038**	0.676	**0.022**	0.067
pH	-0.52	0.31	-0.61	**-0.79**	**0.68**		0.054	**0.022**	0.174	0.098	**0.001**
Ergosterol	-0.01	-0.42	0.47	**0.71**	**-0.77**	-0.62		**0.002**	0.214	**0.006**	**0.022**
Chitinase_V	0.43	-0.26	**0.65**	**0.72**	**-0.66**	**-0.71**	**0.84**		0.128	0.108	**0.004**
Lipase_V	0.26	0.12	0.62	0.26	-0.15	-0.47	0.43	0.52		0.603	0.365
Phosphatase_V	0.14	**0.71**	-0.55	**-0.77**	**0.71**	0.55	**-0.79**	-0.54	-0.19		0.090
ß-glucosidase_V	0.55	-0.44	0.58	**0.70**	-0.60	**-0.87**	**0.71**	**0.82**	0.32	-0.56	

c) Soils of PBSA–soil systems

	Taxa_S_Bac	Taxa_S_Fungi	TOC	TN	C_N	pH	Ergosterol	Chitinase_V	Lipase_V	Phosphatase_V	ß-glucosidase_V
Taxa_S_Bac		**0.019**	0.174	**0.029**	**0.013**	**0.017**	**0.009**	**0.011**	0.855	0.803	0.229
Taxa_S_Fungi	**0.72**		**0.011**	**0.002**	**0.021**	**0.047**	0.079	**0.003**	0.687	0.724	0.197
TOC	-0.47	**-0.76**		**0.011**	0.446	**0.017**	0.405	**0.019**	0.489	0.405	0.074
TN	**-0.68**	**-0.84**	**0.76**		**0.011**	**0.024**	0.067	**0.001**	0.556	0.855	0.098
C_N	**0.75**	**0.71**	-0.27	**-0.76**		0.148	**0.006**	**0.011**	0.777	0.726	0.293
pH	**0.73**	**0.64**	**-0.73**	**-0.70**	0.49		**0.008**	**0.007**	0.093	0.166	**0.003**
Ergosterol	**-0.77**	-0.58	0.30	0.60	**-0.79**	**-0.78**		**0.016**	0.150	0.385	**0.043**
Chitinase_V	**-0.76**	**-0.82**	**0.72**	**0.87**	**-0.76**	**-0.78**	**0.73**		0.214	0.511	**0.009**

Lipase_V	-0.07	-0.15	0.25	0.21	-0.10	-0.56	0.49	0.43		**0.000**	**0.002**
Phosphatase_V	0.09	-0.13	0.30	0.07	0.13	-0.47	0.31	0.24	**0.92**		**0.025**
ß-glucosidase_V	-0.42	-0.45	0.59	0.55	-0.37	**-0.83**	**0.65**	**0.77**	**0.85**	**0.70**	

Table S2 Goodness-of-fit statistics (R^2) of treatment, physicochemical properties, and fungal biomass fitted to the nonmetric multidimensional scaling (NMDS) ordination of enzymes based on Euclidean distance similarity of soils in all treatment. Bold values of *P* and R^2 indicate statistical significances ($p < 0.05$ and $R^2 > 0.7$).

	Enzyme pattern	
**Treatment/ soil physiochemical properties**	$\boldsymbol{R^2}$	***P***
Treatment	0.39	**0.012**
Total organic carbon (TOC)	0.62	**0.003**
Total nitrogen (TN)	0.19	0.182
C:N ratio	0.16	0.264
pH	0.51	**0.004**
PBSA amendment	0.39	**0.008**
N amendment	0.19	0.195
Ergosterol	0.15	0.212
